# Validation of Computer-Adaptive Contrast Sensitivity as a Tool to Assess Visual Impairment in Multiple Sclerosis Patients

**DOI:** 10.3389/fnins.2021.591302

**Published:** 2021-02-23

**Authors:** Sina C. Rosenkranz, Barbara Kaulen, Hanna G. Zimmermann, Ava K. Bittner, Michael Dorr, Jan-Patrick Stellmann

**Affiliations:** ^1^Institut für Neuroimmunologie und Multiple Sklerose, Zentrum für Molekulare Neurobiologie, Hamburg, Germany; ^2^Klinik und Poliklinik für Neurologie, Universitätsklinikum Hamburg-Eppendorf, Hamburg, Germany; ^3^Experimental and Clinical Research Center, Max Delbrück Center for Molecular Medicine and Charité – Universitätsmedizin Berlin, Corporate Member of Freie Universität Berlin, Humboldt-Universität zu Berlin, and Berlin Institute of Health, Berlin, Germany; ^4^NeuroCure Clinical Research Center, Charité-Universitätsmedizin Berlin, Corporate Member of Freie Universität Berlin, Humboldt-Universität zu Berlin, and Berlin Institute of Health, Berlin, Germany; ^5^College of Optometry, Nova Southeastern University, Fort Lauderdale, FL, United States; ^6^Department of Ophthalmology, Stein Eye Institute, University of California, Los Angeles, Los Angeles, CA, United States; ^7^Adaptive Sensory Technology, Lübeck, Germany; ^8^APHM, Hôpital de la Timone, CEMEREM, Marseille, France; ^9^Aix Marseille Université, CRMBM, CNRS UMR 7339, Marseille, France

**Keywords:** qCSF, AULCSF, precision, repeatability, discrimination, multiple sclerosis, vision

## Abstract

**Background:**

Impairment of visual function is one of the major symptoms of people with multiple sclerosis (pwMS). A multitude of disease effects including inflammation and neurodegeneration lead to structural impairment in the visual system. However, the gold standard of disability quantification, the expanded disability status scale (EDSS), relies on visual assessment charts. A more comprehensive assessment of visual function is the full contrast sensitivity function (CSF), but most tools are time consuming and not feasible in clinical routine. The quantitative CSF (qCSF) test is a computerized test to assess the full CSF. We have already shown a better correlation with visual quality of life (QoL) than for classical high and low contrast charts in multiple sclerosis (MS).

**Objective:**

To study the precision, test duration, and repeatability of the qCSF in pwMS. In order to evaluate the discrimination ability, we compared the data of pwMS to healthy controls.

**Methods:**

We recruited two independent cohorts of MS patients. Within the precision cohort (*n* = 54), we analyzed the benefit of running 50 instead of 25 qCSF trials. The repeatability cohort (*n* = 44) was assessed by high contrast vision charts and qCSF assessments twice and we computed repeatability metrics. For the discrimination ability we used the data from all pwMS without any previous optic neuritis and compared the area under the log CSF (AULCSF) to an age-matched healthy control data set.

**Results:**

We identified 25 trials of the qCSF algorithm as a sufficient amount for a precise estimate of the CSF. The median test duration for one eye was 185 s (range 129–373 s). The AULCSF had better test–retest repeatability (Mean Average Precision, MAP) than visual acuity measured by standard high contrast visual acuity charts or CSF acuity measured with the qCSF (0.18 vs. 0.11 and 0.17, respectively). Even better repeatability (MAP = 0.19) was demonstrated by a CSF-derived feature that was inspired by low-contrast acuity charts, i.e., the highest spatial frequency at 25% contrast. When compared to healthy controls, the MS patients showed reduced CSF (average AULCSF 1.21 vs. 1.42, *p* < 0.01).

**Conclusion:**

High precision, usability, repeatability, and discrimination support the qCSF as a tool to assess contrast vision in pwMS.

## Introduction

Visual impairment can be one of the major symptoms in multiple sclerosis (MS) patients. Because vision is rated as one of the three most important bodily functions by MS patients, its impairment has a high impact on quality of life (QoL; Balcer et al., 2015; Heesen et al., 2018). Structural pathological changes in the retina are caused not only by acute optic neuritis, but also as the consequence of chronic inflammation, demyelination, and progressive neurodegeneration (Talman et al., 2010). Key pathological features observed in the retinas of MS patients are decreased thickness of the retinal nerve fiber layer (RNFL), ganglion cell layer, and inner plexiform layer (Balcer et al., 2015; Martinez-Lapiscina et al., 2016; Petzold et al., 2017). However, vision outcomes are not yet routinely implemented as a disease monitoring tool in clinical care or clinical trials for MS. This is mainly due to the fact that the available tests either do not sufficiently represent the pathological changes in the central nervous system or are too time-consuming to incorporate into routine clinical practice.

Currently, visual impairment in clinical practice is usually assessed by the Snellen chart, which records high-contrast visual acuity (HCVA) (Gilbert and Hopkinson, 1949). For example, the gold standard for MS disability quantification, the expanded disability status scale (EDSS), relies on HCVA to estimate its visual functioning score. In MS patients, however, low-contrast visual acuity (LCVA) seems to better correlate with the alterations of retinal morphology (Schinzel et al., 2014) and cognitive function (Wieder et al., 2013). LCVA is usually assessed by the low-contrast Sloan letter charts, but the evidence supporting this method is controversial as association with vision-related QoL in MS patients is inconsistent (Mowry et al., 2009; Stellmann et al., 2015b; Sabadia et al., 2016). Sloan LCVA charts usually measure at selected contrast levels [for example 1.25% or 2.5% (Balcer et al., 2012, 2017)] whereas the affected contrast sensitivity changes on an individual basis for different letter sizes. Consequently, contrast sensitivity should be assessed across a range of different letter sizes or spatial frequencies (Balcer et al., 2017), but the most established tools to measure the full contrast sensitivity function (CSF) are time-consuming, unreliable, and not feasible in routine clinical practice (Kalia et al., 2014). The quantitative CSF (qCSF, previously also termed quick CSF) test is a computerized test that uses a Bayesian adaptive method to assess the full CSF, which implicitly includes LCVA and HCVA, quickly but precisely (Lesmes et al., 2010; Dorr et al., 2013).

We recently showed that qCSF could be a useful tool for the assessment of visual function in MS patients as it correlated best with vision-related QoL measured by the National Eye Institute Visual Functioning Questionnaire (NEI-VFQ) (Stellmann et al., 2015b), whereas VA with Sloan charts was not significantly associated with the NEI-VFQ administered to MS patients. However, to establish the qCSF as a diagnostic tool in routine clinical care and research, further validation is necessary in the intended patient population. Here, we studied two independent consecutive MS cohorts in order to (i) optimize the precision and usability and (ii) evaluate repeatability and discrimination of the qCSF in MS patients.

## Materials and Methods

### Patient Cohorts

Between August 2014 until November 2016 we included consecutive patients with a clinically isolated syndrome highly suggestive for MS or patients with a definite MS diagnosis according to the revised McDonald criteria (Polman et al., 2011) for two independent cohorts: (i) *n* = 54 in the *precision* cohort; and (ii) *n* = 44 in the repeatability cohort. Within the precision cohort, we aimed to compare the precision of two different test settings for the qCSF device, namely 25 vs. 50 qCSF trials. The repeatability cohort performed two consecutive qCSF assessments at a single visit to determine within-session variability with the previously established number of trials, and an additional HCVA assessment, which was chosen as the most commonly used standard clinical visual outcome measure. We assessed the typical median test duration for the qCSF method in the repeatability cohort. The cohorts were recruited sequentially without any overlap in the recruitment periods; thus, no patient was included in both cohorts. For an evaluation of the ability of the qCSF to discriminate between normal vision and abnormal vision in MS, we pooled the patients with a RRMS disease course and without any current or previous optic neuritis of both cohorts (*n* = 13 patients, 40 measurements) and compared them to a published normative dataset of 61 age-matched healthy controls (186 measurements because some subjects were tested repeatedly at two visits about a week apart) (Lesmes et al., 2017). The diagnosis of an acute optic neuritis was assessed by an Ophthalmologist and Neurologist. Historical optic neuritis was assessed with clinical history. Previous optic neuritis was excluded to focus on the neurodegenerative changes rather than previous inflammatory damage. All participants with MS were recruited at the MS outpatient clinic, University Medical Centre Hamburg-Eppendorf, and the healthy controls were recruited and tested at the Nova Southeastern University, College of Optometry. All subjects gave their written informed consent prior to any testing. The local ethics committees approved the studies (Ethics Committee of the Board of Physicians in the State of Hamburg, PV4455 for both MS cohorts and the Institutional Review Board at the Nova Southeastern University in Fort Lauderdale, Florida for the healthy controls).

### Data Acquisition

All tests were performed in the same room, under the same daylight illumination, and in the same order for both MS cohorts; the same stipulations applied for the normal healthy controls tested at a different site. Each eye was assessed separately and measured with best habitual correction (e.g., glasses or contact lenses). For the precision analysis, the repeatability and the analysis of the time duration we treated within-subject eyes as independent measurements, for the discrimination analysis we averaged the available measurements per subject. To assess the full CSF, for each of the 25 trials, the qCSF device (Manifold Contrast Vision Meter, Adaptive Sensory Technology) presented three bandpass-filtered Sloan letters of varying size and contrast on a 46-inch computer screen at a viewing distance of 4.5 m (for details see Lesmes et al., 2010; Dorr et al., 2013). The letters were presented until the patient gave a response; hence the test duration depended on the individual being tested. Based on the participant’s responses, the method chose the most informative combination of size and contrast for the next trial. Test duration for the qCSF was calculated by the file timestamps of subsequent tested eyes. This estimation includes changing the eye patch, any break (rest period) requested by the subject, and re-entering test details. For the precision cohort we ran the qCSF with 50 instead of 25 trials. For the repeatability cohort, the HCVA at 5 m (VA 500) was additionally determined with standard Snellen charts presenting nine lines with 1 to 10 letters. The smallest line with less than two mistakes was defined VA500 (possible values in LogMAR: −0.1, 0, 0.2, 0.3, 0.5, 0.6, 0.7, 0.85, and 1). For all MS subjects, the clinical neurological status was assessed by trained neurologists with the EDSS including its functional system subscore for vision (Kurtzke, 1983). [For correlations of area under the log CSF (AULCSF) and CSF acuity to VA500 see Supplementary Figures 1A,B, for correlations of AULCSF with EDSS and disease duration see Supplementary Figures 2A,B].

### Contrast Sensitivity Function Outcomes

The shape of the CSF can be modeled with four parameters: (i) peak spatial frequency, (ii) peak contrast sensitivity, (iii) bandwidth, and (iv) a low-frequency truncation parameter (Lesmes et al., 2010). The qCSF provides not only scalar estimates of these parameters, but their full joint posterior distribution. However, to simplify statistics and facilitate comparison with the point estimates of existing charts, here we calculated and report several scalar features of the CSF: (i) the AULCSF in the range from 1.5 to 18 cycles per degree; (ii) the CSF acuity, the spatial frequency for which sensitivity reaches zero (i.e., 100% contrast is required for recognition); and (iii) a “low-contrast CSF acuity” as an approximate equivalent of low-contrast acuity charts. Because of the different stimuli (bandpass-filtered vs. unfiltered letters) and the different contrast definitions (Michelson vs. Weber contrast), contrast values (typically 1.25% or 2.5% for LCVA charts) are not directly comparable, so we varied the threshold to 2.5% and in 5% steps from 5 to 50%. In our repeatability cohort, best repeatability precision was obtained with the spatial frequency for which sensitivity reached 0.6 (=log10(4), i.e., 25% contrast required for recognition), and we will thus refer to this parameter as “CS4.”

### Data Analysis

Because the ground truth of the actual CSF of an observer is unknown, we estimated the accuracy by calculating the convergence in the precision cohort by running the qCSF for 50 trials. We also calculated the difference of the CSF estimates after 25 and 50 trials (mean bias and mean absolute bias). For calculating the within-visit test-retest repeatability we used the Bland-Altman coefficient of repeatability (COR) (Bland and Altman, 1986). However, standard vision charts have a limited number of values and low discriminant abilities for the continuum of visual functioning. From a conceptual point of view, this leads to a risk of artificially high repeatability at the cost of low sensitivity to detect subtle differences, and restricts the use of COR for comparison of quantized tests (e.g., vision charts) and continuous outcomes, such as AULCSF. In recent work, we therefore have developed a new metric that penalizes test quantization, namely, Mean Average Precision (MAP) (Dorr et al., 2018). This metric assesses how uniquely an individual is identified by their test and repeated test pair: Repeated tests from the same subject should yield the same result, whereas different subjects should typically yield different results. MAP ranks all retests by their similarity to the first subject’s test and an average rank precision over all participants can be computed. A MAP score of 1.0 indicates high precision and resolution, whereas the MAP score approaches zero for a poor test. For the discrimination between healthy controls and MS patients, we calculate a between-group *t*-test on the AULCSF summary statistic.

## Results

### Quantitative CSF Precision and Number of Trials

We have already shown that 25 trials of the qCSF algorithm are sufficient for a good estimate of the CSF in MS patients (Stellmann et al., 2015b). However, greater precision may be obtained by running the adaptive Bayesian procedure for more trials. In the precision cohort, we analyzed the difference of the estimated CSF between 25 and 50 trials by calculating the convergence. Descriptive statistics for the precision cohort are summarized in Table 1.

**TABLE 1:** Baseline characteristics of the precision cohort.

Baseline characteristics (*n*)	Patients (54)
Age, mean (SD), range, years	41 (10), 20–63
Gender, male/female	15/39
EDSS, mean (SD), range	2.39 (1.41), 0–6.5
Time since first symptoms, years (SD), range	8 (8), 0–29
CIS, *n* (%)	10 (18.5)
RRMS, *n* (%)	38 (70.4)
PPMS, *n* (%)	3 (5.5)
SPMS, *n* (%)	3 (5.5)

*SD = Standard deviation; EDSS = Expanded Disability Status Scale; CIS = Clinically isolated syndrome; RRMS = Relapsing remitting MS; SPMS = Secondary progressive MS; PPMS = Primary progressive MS.*

**FIGURE 1:**
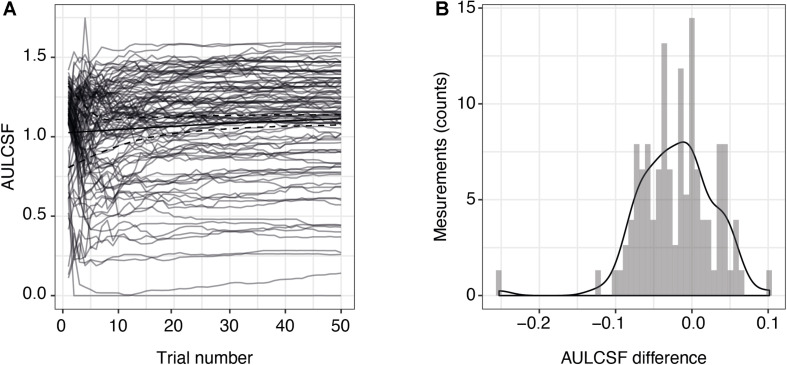
Precision of AULCSF estimate over time. **(A)** AULCSF estimates for each eye (gray lines) (*n* = 105 measurements) and their mean over all measurements (solid black line). The dashed black lines show the mean lower and upper ends of the 68.3% credible interval estimates averaged across all measurements and are therefore indicative of the internal measurement variability (not of variability across measurements). **(B)** Distribution of differences between AULCSF estimates after 25 and 50 trials (*n* = 105 measurements). The *y*-axis shows the absolute number of measurements within each bin of differences. AULCSF = area under the log CSF.

Two of the 54 included subjects with MS were measured monocularly only because they had no remaining vision in one of their eyes. One measurement in one subject had to be excluded due to technical problems. This resulted in a total of 105 qCSF measurements. Figure 1A shows the convergence of the AULCSF estimate over the time course of 50 trials (internal variability, not variability across measurements). For many patients, the first incorrect response leads to a drop in the AULCSF estimate, which then recovers after the first 5–10 trials. After 25–30 trials, the mean AULCSF estimate and the credible interval width have mostly converged with a small but statistically significant difference between the AULCSF estimate after 25 and after 50 trials (Figure 1B); (for 105 measurements mean = −0.02 log10 units, SD = 0.049; 95% CI (−0.03, −0.01), *one-sample t-test t* (104) = −4.34, *p* < 0.001). In line with previous work and the negligible benefit above 25 trials compared to the cost of additional test time, we performed subsequent testing and analyses with 25 trials.

### Quantitative CSF Repeatability

**FIGURE 2:**
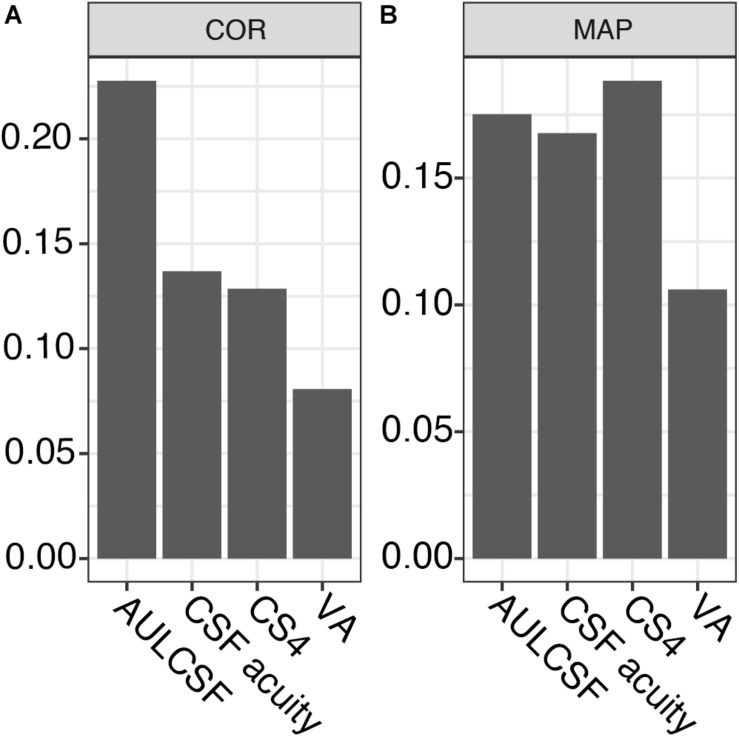
Repeatability metrics for AULCSF, CSF acuity, CS4, and VA500. **(A)** Coefficient of repeatability (only for the same feature, lower values correspond to greater precision). **(B)** Mean Average Precision (higher values correspond to greater precision, independent of test feature). AULCSF = area under the log CSF; CSF acuity = visual acuity of contrast sensitivity function; CS4 = CSF acuity at 25% contrast; VA = visual acuity; COR = coefficient of repeatability; MAP = Mean Average Precision.

Next, we assessed the individual within-visit reliability of two repeated tests in the repeatability cohort of MS patients. We included 44 subjects with one who was measured in one eye only. This resulted in 87 pairs of test and retest measurements; one eye was excluded from the CS4 calculation because its contrast sensitivity was too low (no stimulus size could be recognized at 25% contrast). Table 2 shows the baseline descriptive statistics of this cohort. Numerically, AULCSF had the highest (“least repeatable”) COR in comparison to VA500 based on standard charts, CSF acuity, and CS4 (0.23 vs. 0.08, 0.14, 0.13, respectively, Figure 2A; for Bland-Altman plots, see Supplementary Figures 3A–D). However, the test score ranges are not directly comparable and paper charts strongly quantize results (see Supplementary Figure 3D), i.e., lack resolution. Using the novel metric MAP that penalizes coarse quantization, the AULCSF, high-contrast CSF acuity and low-contrast CS4 had better repeatability and precision (higher MAP values) than VA500 (0.18, 0.17, 0.19. vs. 0.11, respectively, Figure 2B).

**TABLE 2:** Baseline characteristics of the repeatability cohort.

Baseline characteristics (*n*)	All patients (44)
Age, mean (SD), range, years	41 (9.7), 22–58
Gender, male/female	13/31
EDSS, mean (SD), range	2.49 (1.73), 0–6.5
Time since first symptoms, years (SD), range	9.2 (9.1), 0–36
Visual acuity,% (SD), range	83.2 (25.3), 10–125
CIS, *n* (%)	5
RRMS, *n* (%)	25
PPMS, *n* (%)	1
SPMS, *n* (%)	2

*SD = Standard deviation; EDSS = Expanded Disability Status Scale; CIS = Clinically isolated syndrome; RRMS = Relapsing remitting MS; SPMS = Secondary progressive MS; PPMS = Primary progressive MS.*

### Quantitative qCSF Test Duration

**FIGURE 3:**
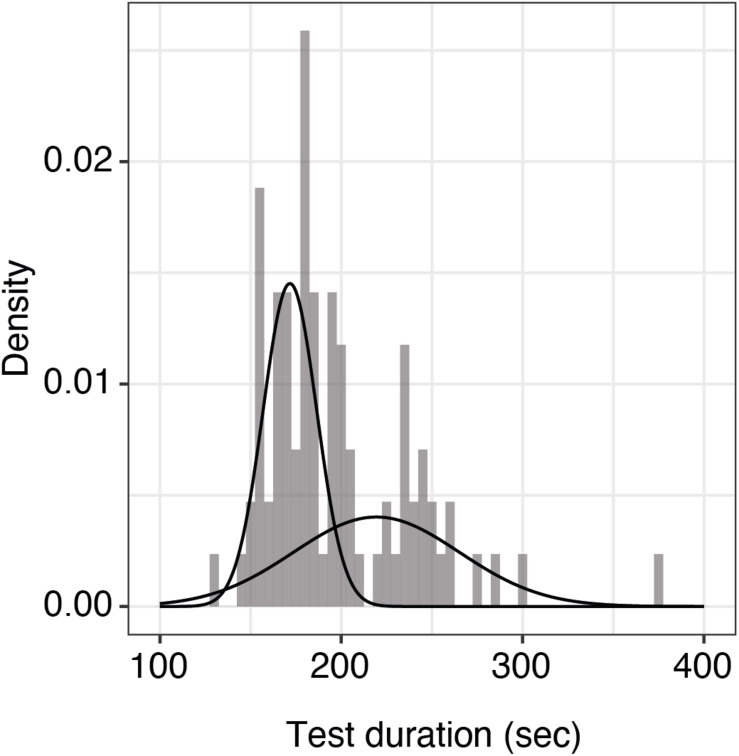
Histogram of test durations per eye including all preparations. Shown here is the distribution of qCSF test duration per eye (*n* = 86 measurements) including all preparations. The raw data were best fit by a mixture of two Gaussian distributions with means of 172 and 219 s, respectively. The overall median test duration per eye is 185 s.

Test duration is an important factor for clinical usability. We looked at the duration between subsequent tests of 25 trials each for alternating eyes in the repeatability cohort (43 patients, the patient with only one measured eye was excluded). As the data were not normally distributed, we fit a mixture of two Gaussian distributions (mean 172 and 219 s; standard deviation 17 and 10 s, respectively). Median test duration per eye including all preparation, such as changing the eye patch and a break between eyes, was 185 s (5th and 95th percentiles were 154 and 260 s, respectively, Figure 3).

### Discriminative Power of the qCSF in MS

**FIGURE 4:**
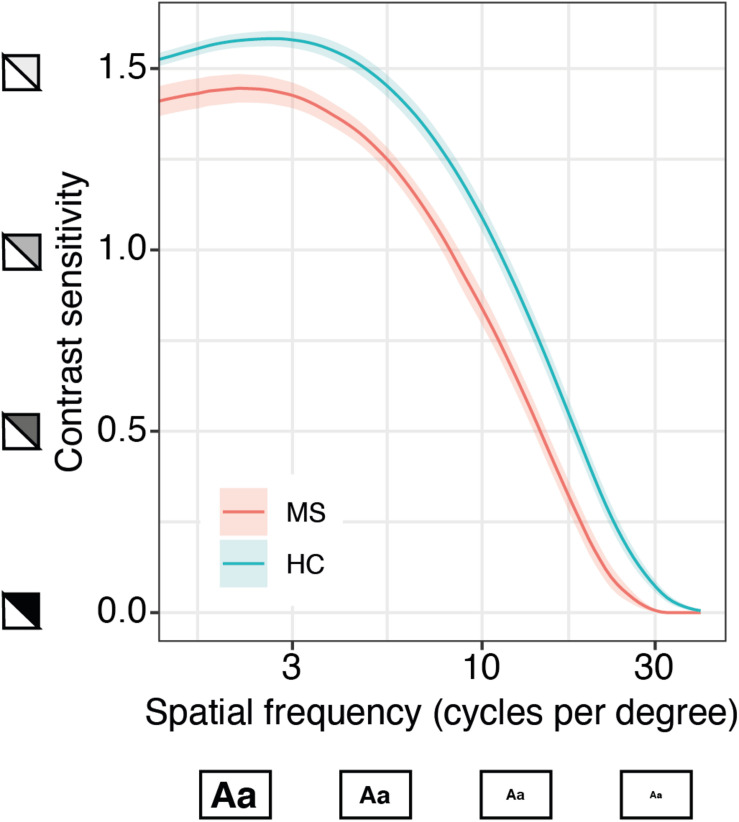
Quantitative contrast sensitivity function (qCSF)-examination in MS patients compared to age-matched healthy controls (HC). Curves show mean contrast sensitivity function (CSF) in MS patients (*n* = 13, based on patient-specific averages of a total of 40 measurements) and HC (*n* = 61, 186 measurements) ± SEM, *x*-axis represents logarithmic spatial frequencies, i.e., decreasing size of the letters; *y*-axis represents logarithmic sensitivity to decreasing contrast.

We further investigated whether the qCSF could serve as a diagnostic instrument in MS and could discriminate these patients from healthy controls. For this aim, we pooled all qCSF measurements from both MS cohorts (precision and repeatability cohort), excluded all patients with a previous optic neuritis or an unknown visual symptom history and compared them to a normative data set of age-matched healthy normal controls (*n* = 186 measurements from 61 subjects, age range 20–59 years). On average, MS subjects (mean EDSS 2.65; SD 1.62) had reduced CSF results when compared to healthy controls (mean AULCSF after averaging measurements per subject 1.21 vs. 1.42, 95% CI of group differences (0.12, 0.3), Welch’s *t* (36.28) = 4.77, *p* < 0.001). The most pronounced difference is seen on the top left side of the curve indicating a larger gap of performance for large and medium-sized letters at low contrast (Figure 4).

## Discussion

Precise, reliable assessment of visual function is an important part of disability quantification in MS (Balcer et al., 2015; Heesen et al., 2018). However, currently available tools have several limitations and their impact on clinical decisions is probably negligible. Here, we provide important feasibility and reliability information about a new outcome to assess visual impairment in MS. The qCSF method shows higher precision than the current standard of care (VA500) without major burden to the patient or clinician. The qCSF has previously been compared to standard vision outcomes in MS patients, namely HCVA and LCVA, and already has demonstrated higher correlation with visual quality of life from the MS patient’s perspective than HCVA or LCVA (Stellmann et al., 2015b).

In a first consecutive cohort, we aimed to define a reliable trade-off between duration and precision of the qCSF computer-adaptive algorithm. Conceptually, precision of an estimate increases as a function of time as the impact of outliers and lapses decreases with the number of trials. However, long test duration directly reduces the feasibility and acceptance in clinical care and trials. For example, longer walking tests of 2 or 6 min for several years already have been considered to replace the MS standard of a timed 25 foot walk (which usually takes less than 1 min), but implementation of such outcomes is still rare (Gijbels et al., 2012; Stellmann et al., 2015a). We found that 25 trials were sufficient for a reliable convergence of the algorithm, and a longer test duration did not change the estimate of the AULCSF substantially. This is in line with a previous study that showed that 25 trials of the qCSF were enough to demonstrate loss of visual function in diabetic subjects with and without retinopathy compared to healthy controls (Joltikov et al., 2017). However, longer test durations might still be necessary if other, potentially noisier features than the summary statistic AULCSF were of interest.

The second aim of our study was to evaluate the repeatability performance of the qCSF method in MS patients. First, we found that the visual function estimate did not differ substantially between two separate assessments in each individual, i.e., we found high stability of the method or high intra-individual reliability. This held true for both the evaluated parameters of the qCSF and for visual acuity VA500. However, care must be taken not to confound the commonly reported COR as a tool to compare tests with different outputs. The smaller range and the strong quantization of VA500 scores led to a seemingly excellent COR. However, repeatability (or precision) is only a necessary but not sufficient condition for the more important quality of a biomarker, which is the sensitivity to detect changes in the underlying signal. In the absence of a ground truth of visual change, we hypothesize that different individuals differ in their visual function, and the MAP metric quantifies the discriminant ability of a test for each individual in comparison to the whole group. Here, in a reversal of the ranking based on the COR, the summary statistic AULCSF provided higher discriminant ability than VA500 and CSF acuity.

Similar to low-contrast Sloan VA charts, we further calculated the cut-off frequency for stimuli presented at 25% contrast (CS4), and found that this parameter was even more precise than the AULCSF. In principle, the combination of several parameters, or the distributions thereof, might provide even further precision gains. This also demonstrated the advantage of estimating the full CSF versus paper charts that are limited to a fixed contrast level (HCVA or LCVA) or fixed spatial frequency (e.g., Pelli-Robson CS chart) (Pelli and Bex, 2013). In addition to the summary statistic AULCSF, we were able to calculate cut-off frequencies for a large number of contrast levels, in order to select the parameter with the highest test-retest precision. In general, different ocular or neurological pathologies might affect vision differently at different stages, and the most informative part of the CSF may therefore differ across individuals or diseases. For example, low vision patients may experience floor effects at low contrast levels, which was the case for one MS patient at our 25% contrast criterion. Testing the entire CSF thus solves the conundrum of knowing where to test in advance.

Furthermore, we also assessed typical test duration. With an average of about 3 min, results are in agreement with previous qCSF data from healthy controls (Dorr et al., 2017). Taken together with the comparable COR (Lesmes et al., 2017), this implies that mildly to moderately impaired MS patients do not need specialized test protocols because they are as fast and precise, albeit at a lower performance level, than healthy controls. Notably, the test duration for the qCSF in our study seems comparable to Sloan charts in previous studies. The literature reports a test duration of 10–15 min for a complete monocular and binocular testing at two contrast levels for the Sloan charts (Balcer et al., 2017).

The descriptive analysis comparing average CSF estimates from MS and healthy controls indicates a worse performance over the complete frequency range with marked impairment of sensitivity to lower contrast levels. This finding in our study is consistent with reports for Sloan charts, that provide best discriminant abilities for MS with 1.25 and 2.5% low contrast VA charts. While 0.6% seems to be biased by a floor effect, 5% charts are already limited by a raising ceiling effect (Balcer et al., 2017). The qCSF approach avoids test restrictions at selected contrast levels, which may differ with disease progression, or spatial frequencies as the full CSF is estimated. The current main outcome, AULCSF, might thus be sensitive to differentiate patients from healthy controls as subtle differences over the complete CSF might sum up to a significant difference of the AULCSF. However, a sufficiently powered future head-to-head comparison of matched individuals tested under the same conditions might reveal even more discriminatory features of the CSF. Moreover, to establish qCSF as a diagnostic tool for MS, prospective studies are needed to determine sensitivity for change over time and define meaningful clinically relevant cut-offs.

The main limitation of the current study was the lack of comparison with Sloan VA charts in MS patients. However, we aimed to keep the burden for recruited patients low and decided to contrast our findings only with the clinical and EDSS standard, which is HCVA with a Snellen chart. Interestingly, repeatability data for Sloan charts in MS patients have not been previously published, although a good intra-rater agreement has been reported (ICC 0.86–0.95) (Balcer et al., 2000). It should also be noted that the data from healthy controls were collected in a different clinic under slightly different conditions, at a viewing distance of 400 vs. 450 cm. However, these subtle factors likely did not substantially affect the rather large (qualitative) difference in qCSF on average that we observed between MS and healthy controls.

Taken together, qCSF provides excellent test characteristics and has already been linked to visual quality of life in MS. Our findings indicate that the further evaluation of the qCSF method in longitudinal studies as an outcome measure for MS seems promising. These studies should also include comparisons to the Sloan VA charts.

## Data Availability Statement

The raw data of all MS patients supporting the conclusions of this article will be made available by the authors, without undue reservation.

## Ethics Statement

The studies involving human participants were reviewed and approved by Ethics Committee of the Board of Physicians in the State of Hamburg and the Institutional Review Board at the Nova Southeastern University in Fort Lauderdale, Florida. The patients/participants provided their written informed consent to participate in this study.

## Author Contributions

J-PS conducted and designed the experiments. AKB designed the study involving healthy controls. SCR, MD, and J-PS analyzed the data and wrote the manuscript. BK analyzed the data. HZ and AKB supervised the study and wrote the manuscript. All authors contributed to the article and approved the submitted version.

## Conflict of Interest

MD holds equity in and employment by Adaptive Sensory Technology and holds patents related to the qCSF. The qCSF device was provided to INIMS free of charge; no further compensation was granted. HZ received research grants from Novartis and speaking honoraria from Bayer Healthcare, unrelated to this study. The remaining authors declare that the research was conducted in the absence of any commercial or financial relationships that could be construed as a potential conflict of interest.
