# Supplementary material for: Validation of Computer-Adaptive Contrast Sensitivity as a Tool to Assess Visual Impairment in Multiple Sclerosis Patients
Source: Front Neurosci. 2021 Feb 23;15:591302. doi: 10.3389/fnins.2021.591302 (PMC7940823; doi:10.3389/fnins.2021.591302)
Supplement: Supplementary file 1 [file Data_Sheet_1.pdf]

Supplementary Material

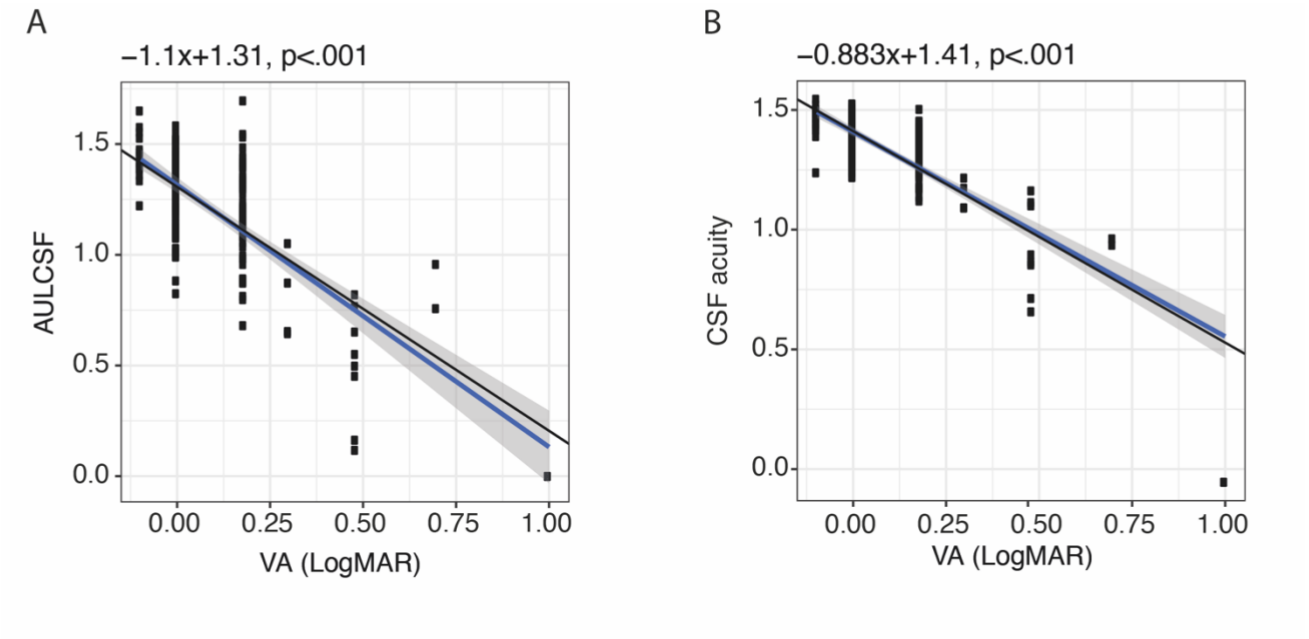

Supplementary Figure 1. Correlation between AULCSF (A) and CSF acuity (B) and VA500. In a linear mixed-effects model (n=44 patients, measurements of the *repeatability cohort)*, AULCSF (**A**, p<0.001) and CSF acuity (**B**, p<0.001) were significantly correlated with VA500. The black line and numerical results represent the results from the linear mixed-effects model; for comparison, the blue line and confidence interval (shaded area) represent the results from a linear regression that considers all measurements as independent.

AULCSF= area under the log CSF; CSF acuity= visual acuity of contrast sensitivity function; VA= visual acuity.

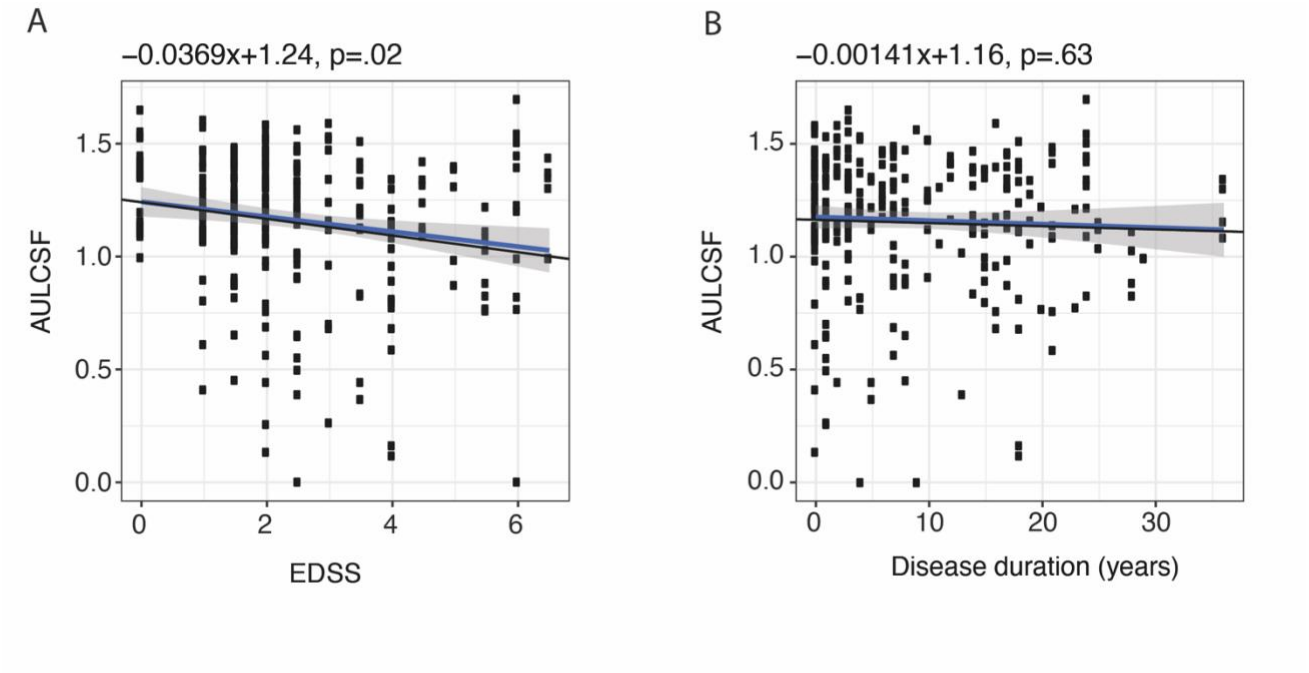

Supplementary Figure 2. Correlation between AULCSF and EDSS (A) and disease duration (B).

In a linear mixed-effects model with patient as random effect (n=98 patients, measurements of both MS cohorts pooled including patients with optic neuritis), AULCSF was significantly correlated with EDSS (**A**; Welch's t (104.8) =-2.37, p=.02), but not with disease duration (**B**; Welch's t (111.2) =-0.49, p=.63). The black line and numerical results represent the results from the linear mixed-effects model; for comparison, the blue line and confidence interval (shaded area) represent the results from a linear regression that considers all measurements as independent.

AULCSF= area under the log CSF.

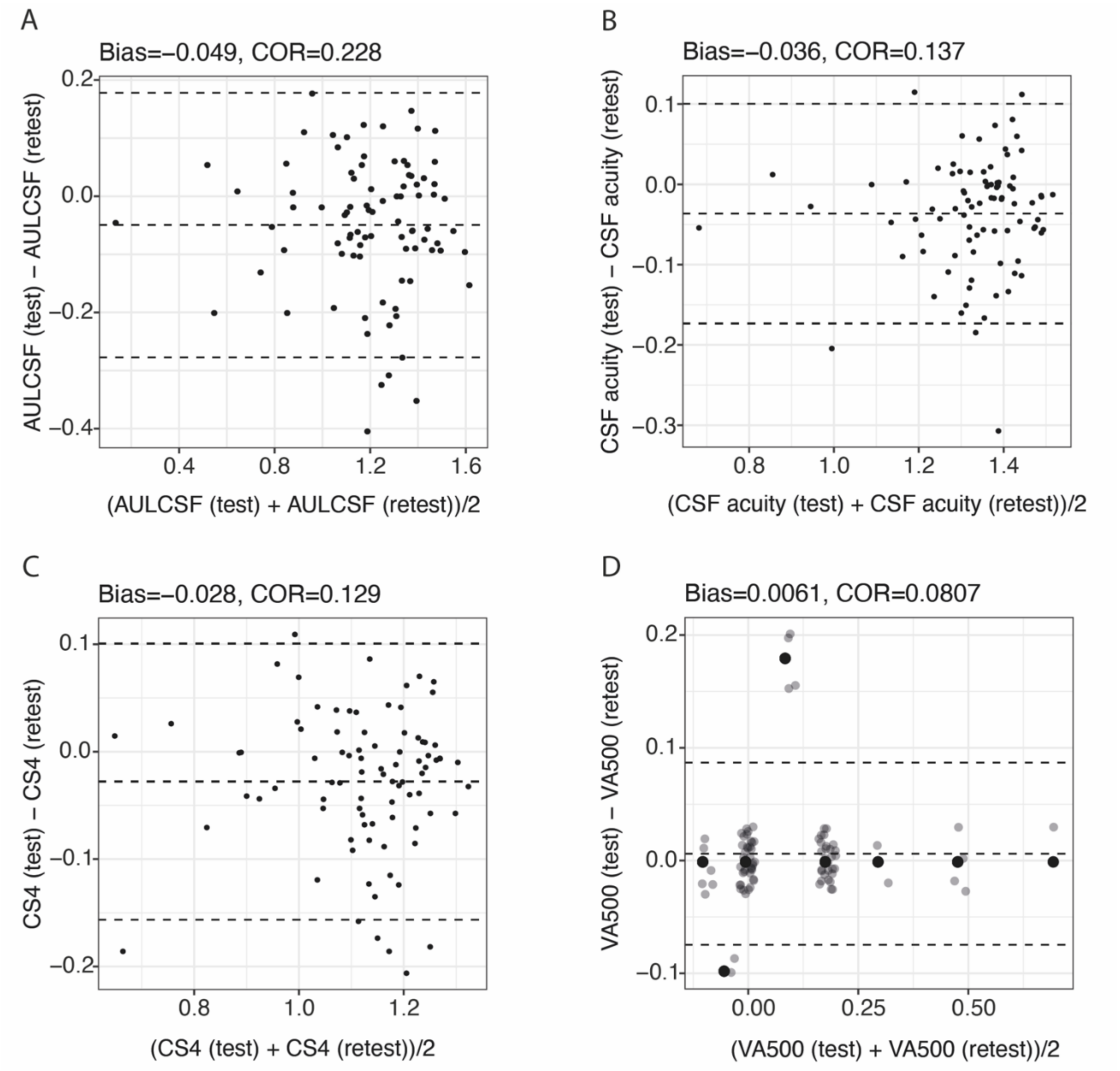

Supplementary Figure 3. Agreement of two measurements for a) AULCSF, b) CSF acuity, c) CS4 and d) VA500. The small grey point clouds in d) are jittered to indicate how many measurements are represented by each of the big black points.

AULCSF= area under the log CSF; CSF acuity= visual acuity of contrast sensitivity function; CS4= CSF acuity at 25% contrast; VA= visual acuity; COR= coefficient of repeatability.
